# Development of the sickle Pan-African research consortium registry in Tanzania: opportunity to harness data science for sickle cell disease

**DOI:** 10.3389/frhem.2023.1040720

**Published:** 2023-04-10

**Authors:** Daniel Kandonga, Raphael Zozimus Sangeda, Upendo Masamu, Eliah Kazumali, Agnes Jonathan, Michael Msangawale, Winfrida Kaihula, Julieth Rwegalulila, Jesca Ondego, Hilda J. Tutuba, Joyce Ndunguru, Emmanuela E. Ambrose, Benson R. Kidenya, Mbonea Yonazi, Irene Kyomugisha, Wilson Mupfururirwa, Mario Jonas, Victoria Nembaware, Gaston Kuzamunu Mazandu, Andre Pascal Kengne, Ambroise Wonkam, Julie Makani, Emmanuel Balandya

**Affiliations:** 1 Sickle Cell Programme, Muhimbili University of Health and Allied Sciences, Dar es Salaam, Tanzania,; 2 Department of Pharmaceutical Microbiology, Muhimbili University of Health and Allied Sciences, Dar es Salaam, Tanzania,; 3 Statistics Department, Eastern Africa Statistical Training Centre, Dar es Salaam, Tanzania,; 4 Department of Paediatrics and Child Health, Catholic University of Health and Allied Sciences, Mwanza, Tanzania,; 5 Department of Biochemistry and Molecular Biology, Catholic University of Health and Allied Sciences, Mwanza, Tanzania,; 6 Department of Hematology and Blood Transfusion, Muhimbili National Hospital, Dar es Salaam, Tanzania,; 7 Department of Pathology, Division of Human Genetics, Faculty of Health Sciences, University of Cape Town, Cape Town, South Africa,; 8 Non-Communicable Diseases Research Unit, South African Medical Research Council, Cape Town, South Africa,; 9 Department of Genetic Medicine, Johns-Hopkins University School of Medicine, Baltimore, MD, United States,; 10 SickleInAfrica Clinical Coordinating Centre (CCC), Muhimbili National Hospital, Dar es Salaam, Tanzania,; 11 Department of Physiology, Muhimbili University of Health and Allied Sciences, Dar es Salaam, Tanzania

**Keywords:** sickle cell disease, Tanzania, registry, case report form, REDCap, data science

## Abstract

**Background:**

Sickle cell disease (SCD) is a severe hereditary form of anemia that contributes between 50% and 80% of under-five mortality in Africa. Eleven thousand babies are born with SCD annually in Tanzania, ranking 4^th^ after Nigeria, the Democratic Republic of Congo and India. The absence of well-described SCD cohorts is a major barrier to health research in SCD in Africa.

**Objective:**

This paper describes the Sickle Pan African Consortium (SPARCO) database in Tanzania, from the development, design of the study instruments, data collection, analysis of data and management of data quality issues.

**Methods:**

The SPARCO registry used existing Muhimbili Sickle Cell Cohort (MSC) study case report forms (CRF) and later harmonized data elements from the SickleInAfrica consortium to develop Research Electronic Data Capture (REDCap) instruments. Patients were enrolled through various strategies, including mass screening following media sensitization and health education events during World Sickle Cell Day each June and the SCD awareness month in September. Additional patients were identified through active surveillance of previously participating patients in the MSC.

**Results:**

Three thousand eight hundred patients were enrolled between October 2017 and May 2021. Of these, 1,946 (51.21%) were males and 1,864 (48.79%) were females. The hemoglobin phenotype distribution was 3,762 (99%) HbSS, 3 (0.08%) HbSC and 35 (0.92%) HbSb +thalassemia. Hemoglobin levels, admission history, blood transfusion and painful events were recorded from December 2017 to May 2021.

**Conclusion:**

The Tanzania SPARCO registry will improve healthcare for SCD in Africa through the facilitation of collaborative data-driven research for SCD.

## 1 Introduction

Sickle cell disease (SCD) is an inherited disorder of the hemoglobin molecule of the red blood cells, causing them to change into a sickle or crescent shape in areas with low oxygen tension (1). Clinical manifestations begin in the early years of life and generally include anemia, repeated infections, and periodic pain episodes. SCD is most prevalent in malaria-endemic areas in the tropics, with poor health outcomes due to a lack of resources, resulting in high mortality among patients below five years of age (2). Out of the estimated 300,000 annual live births with SCD globally, over 75% are in Sub-Saharan Africa (SSA) (3). In 2050, the annual births of individuals with SCD is projected to reach 400,000 globally, with 85% born in Sub Saharan Africa (SSA) (4). This is why it is critical for proven interventions such as early diagnosis through newborn screening and comprehensive care, including access to penicillin prophylaxis, hydroxyurea, and folic acid should be made available in Africa to reduce mortality and prolong the life of patients (4). In Africa, there are few comprehensive databases for SCD patients that can be said to monitor clinical care (5). Furthermore, the registries could be resources for supporting collaborative research through a collection of longitudinal data on patients to help understand their experiences and clinical outcomes of the disease.

In Tanzania, over 11,000 children are born annually with SCD (4). The Sickle Cell programme (SCP), based at Muhimbili University of Health and Allied Sciences (MUHAS) in Tanzania, established the Muhimbili Sickle Cohort (MSC), which is one of the largest cohorts of SCD patients globally, with 5,476 patients (6). Established in 2011 as a research project funded by the Wellcome Trust, the programme has expanded and established seven specialized SCD clinics by May 2021. Five in the Dar es Salaam region, one in Pwani region and another one in the Mwanza region. The presence of registries with quality data can improve patient management as well as the design of future research. Although, MSC registered more than 5,000 patients within the first ten years (7, 8). The design of the database did not allow utilization for extensive collaborative studies due to two limitations. First, data integration and querying are inherently complex for users to extract information due to several inconsistencies and redundancies. Second, genotype to phenotype associations were not easily supported. Recognizing the need for an interactive registry, MUHAS applied for a collaborative grant on “SCD in sub-Saharan Africa: Collaborative Consortium” (RFA-HL-17–006, 2021b by the National Institutes of Health) and was awarded grant no U24HL135881 (2017–2021) for The Sickle Pan African Research Consortium (SPARCO) which was coordinated from a hub at MUHAS and had two additional sites, at the University of Abuja, Abuja, Nigeria, and the Kwame Nkrumah University of Science and Technology (KNUST), Kumasi, Ghana. The objective of SPARCO (2017–2021) was to establish a uniformly consented database of patients with SCD, with the target to enroll 13,000 patients (Tanzania = 4,000, Ghana = 3,000, Nigeria = 6,000). The University of Cape Town, Cape Town, South Africa, was the successful applicant for the data coordinating center (Sickle Africa Data Coordinating Center – SADaCC) (9). In addition, SPARCO aimed to develop research capacity for SCD through a multidimensional approach that addresses infrastructure, education & training, provision of longitudinal research data, and the translation of research into practice.SPARCO has now grown to include Mali,Uganda, Zambia and Zimbabwe as countries under the consortium. This platform provides a unique opportunity to harness data science for research, including future efforts toward the cure of SCD. These registry aimed to translate research into healthcare and health outcomes through SickleinAfrica registry which includes mult-country registry with different countries in Africa that follow ethical and social concerns (10).

The Research Electronic Data Capture (REDCap) (11, 12) is an accepted tool for sharing data across platforms and geographical boundaries (13), and was the preferred platform for the SPARCO electronic SCD registry. This paper aims to share experiences for enrolling SCD patients into the SPARCO registry in Tanzania. The Ghana and Nigeria SPARCO sites shared their experiences in separate publications (14, 15). Results from this paper could be helpful to other SSA countries sharing similar challenges that are trying to establish new registries and databases for SCD. In addition, this could also be a call for Tanzania SCD stakeholders to harmonize SCD data across the country.

## 2 Materials and methods

### 2.1 Study design

An observational study was conducted at the Sickle Cell Programme, MUHAS, through the SPARCO project. The Sickle Cell Programme (SCP) was established through collaboration between the MUHAS, the oldest and largest biomedical university in Tanzania, and Muhimbili National Hospital (MNH). MNH is the national referral hospital and teaching hospital for MUHAS. MNH is located in Dar-es-Salaam along the eastern coast of Tanzania. In 2017, through SPARCO, the SCP expanded its services to five other clinics to regional referral Hospitals in the Dar es Salaam region (16) and later expanded to other two subsites Bagamoyo District Hospital in Pwani region and Bugando Medical Centre in Mwanza region to make seven enrollment sub-sites. The services provided at the SCD clinics include health education, consultation and enrollment of patients diagnosed with SCD. Patients receive hydroxyurea, penicillin prophylaxis and folic acid. Usually, the clinics operate twice a week or once a week for the district hospitals.

### 2.2 Sample size

All diagnosed SCD patients between October 2017 and May 2021 were eligible for enrollment into the registry. New and previously enrolled patients from the MSC were also included in the study after consenting to be registered in the SPARCO registry. Case report forms (CRF) included data elements with socio-demographic details (name, marital status, gender, contact information), management details (hydroxyurea, penicillin prophylaxis, folic acid, anti-malarial and pneumococcal vaccine), diagnosis (HbSS, HbSC, HbB + Thalassemia), and clinical visits (pain episodes, admission, blood transfusion, hemoglobin level, use of health insurance) for the first and follow-ups. Alkaline cellulose acetate electrophoresis or high-performance liquid chromatography (HPLC) were used as confirmatory tests for SCD.

### 2.3 Registry development and study data element

The site used previous MSC study data elements to obtain the initial data elements for recruiting patients during the early months of enrollment. The base data elements were then modified by consulting the harmonized consortium data elements through SCD ontology (17, 18) which support a uniform database for SCD in Africa under the coordination of SADaCC. The final data elements were then developed into REDCap (11, 12) to startenrollment. In the second round in 2019, additional data elements for the follow-up visits were added. Data quality algorithms were applied to ensure the registry captured the required data. A total of 11 standard operating procedures (SOP’s) have been developed, of which five were adopted from SADaCC.

### 2.4 Consenting process

SPARCO Tanzania adopted consent forms developed by the SickleInAfrica consortium (10). It translated these into the Swahili language, the native and most spoken language in Tanzania, for ease of understanding by patients during enrollment. Patients above 18 years of age were given informed consent to sign. Those aged 7 to 18 years signed the assent form, followed by an additional written consent form for their parents/caregivers to sign. Parental/caregiver consent was also obtained for patients below seven years of age. Patients who did not consent were not enrolled in the registry.

### 2.5 The use of Mobile applications for data collection

In year three of the project, SPARCO Tanzania site started using the REDCap mobile App to capture patients’ data at the sub-sites. Online and offline methods were employed using tablets to ensure timely data collection and improvement in data quality using the data quality features built into the REDCap tool. Regular training of the data managers, data coordinators, data clerks and clinicians was done to ensure proper use of electronic devices at the enrolment sites. Clinicians and data clerks were trained on mobile data capture and adhering to technical standards in enrolling patients through the REDCap Mobile App.

### 2.6 Data quality assurance processes and backup

Data quality served to be important in building blocks towards achieving SPARCO aims. The registry uses REDCap inbuilt rules to restrict data types, range checks, uniform data entry and proper branching logic during the design phase of the registry. Using barcodes on health passports ensures each patient owns a unique registry number, preventing discrepancies. The site developed SOP’s which cover manual review of newly entered data, running data quality rules and reviewing reports. The process was done every week to ensure the quality of the data collected. The data were then backed up to the local server within SCP by considering keeping copies of the registry aside from the daily automated backup system from the server, which is done online.

### 2.7 Patient recruitment and enrollment strategies

Patients recruited into the registry included those who attended outpatient SCD patients at the enrolment sites through self-referral or after referral by attending physicians from the pediatric or adult medical services. Further, patients diagnosed with SCD through mass screening events were referred to nearby SCD clinics for enrolment into the registry. We also took advantage of known patients previously enrolled in the MSC database to conduct active surveillance *via* phone calls and encourage patients to attend nearby SCD clinics for recruitment into the SPARCO database (8). Data was collected by trained clinicians and data collectors. Data was collected directly from patients/caregivers and was subsequently entered into the REDCap database. Frequent training sessions and site visits were conducted to ensure uniform and quality data collection. Patients were enrolled through various strategies, including mass screening following media sensitization and health education events during World Sickle Cell Day each June and the SCD awareness month in September. Additional patients were sourced through active surveillance of previously enrolled patients in the MSC.

### 2.8 Data analysis

SPPS version 23.0 was used for analysis. Categorical variables were summarized in frequencies and percentages. The haemoglobin level were categorized into severe anemia (haemoglobin below 5g/dl), moderate anemia(haemoglobin 5–8g/dl) and mild anemia (haemoglobin above 8g/dl). The significance of difference in distribution of age categories across gender, SCD-Phenotype, recruitment hospitals, drug management, clinical events and health insurance use was compred using Pearson’s chi-square test. P-values of less than 0.05 were considered statistically significant. Microsoft excel software 2019 Map tool was used to draw the map of Tanzania and its respective content.

## 3 Results

**FIGURE 1:**
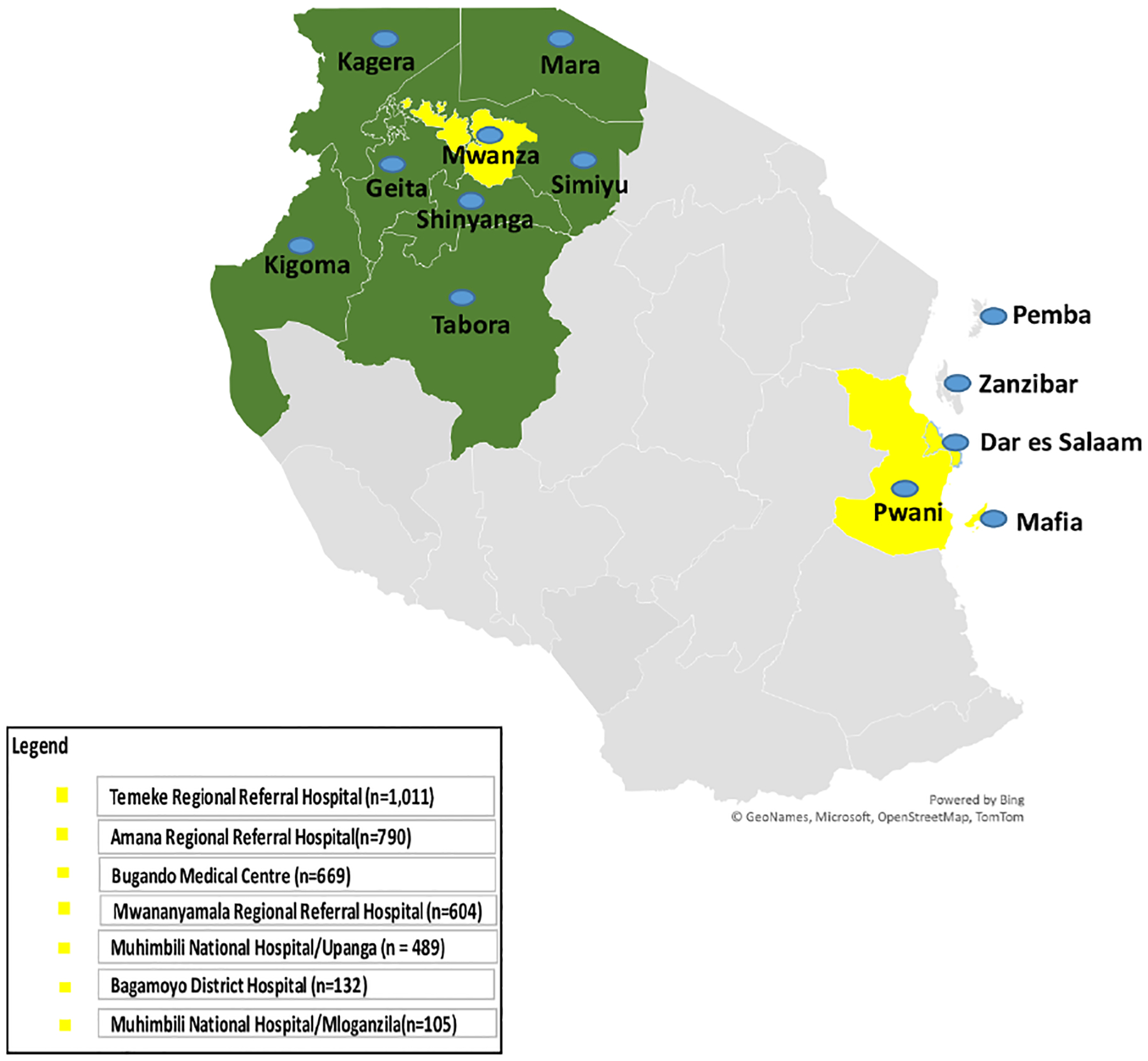
Map of Tanzania showing regions where Sickle Pan-African Research Consortium (SPARCO) sickle cell clinics are located (enrollment sites - in yellow) and regions that refer patients to enrollment sites (catchment area - in green) for 2017 to 2021.

**FIGURE 2:**
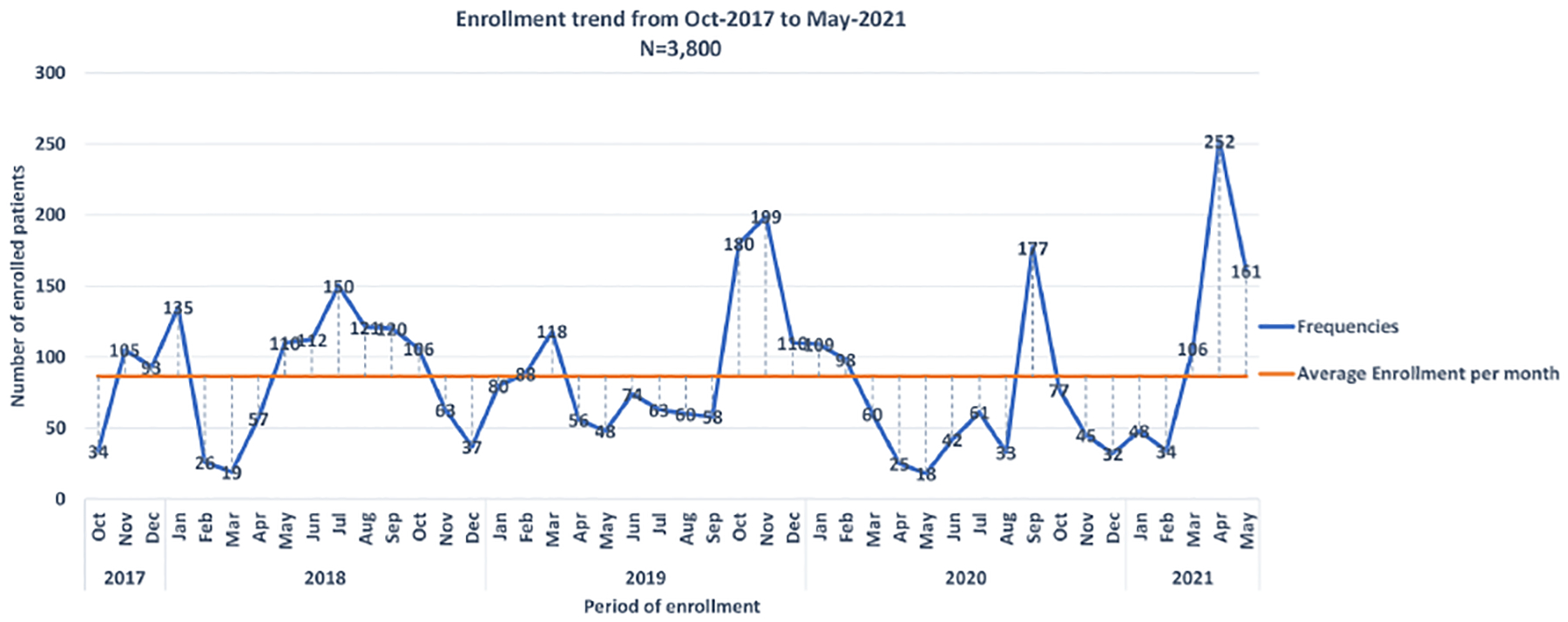
Monthly enrollment trends into the into Sickle Pan African Research Consortium (SPARCO) at Tanzania site between 2017 and 2021.

The SPARCO Tanzania site enrolled 3,800 SCD patients from October 2017 to May 2021, 95% of the targeted 4,000 patients. Registry enrollment trends varied throughout the four years of enrollment. Patients were enrolled from seven outpatient clinics in Dar es Salaam, Pwani and Mwanza regions (Figure 1). More male patients were enrolled into the registry compared to female patients (1,946 (51.2%) versus 1,854 (48.8%). There were 3,095 (81.4%) children below the age of 18 and the remaining 705 (18.6%) were 18 years and above. The lowest peak was observed in March 2018and May 2020 with 19 and 25 patients enrolled respectively. In April 2021, highest peak was observed with 252 patients enrolled (Figure 2). Temeke Regional Referral Hospital enrolled 1,011 (26.6%) patients, which was the highest number compared to other hospitals (Table 1).

Of the 3,800, 3,762 (99%) patients had HbSS phenotype, 3 (0.1%) were confirmed to have HbSC and 35 (0.9%) had HbS beta-plus thalassemia. The proportion of participants with respect to the important clinical events is reported at; painful episodes (4.4%), hospitalization history (1.1%), blood transfusion (1.7%) and severe anemia (45.0%) (Table 2). The use of health insurance was reported in 248 (27.6%) of the respondents. There was no statistically significant difference in age group and the different clinical events or accessibility to the health insurance service (Table 2).

## 4 Discussion

This comprehensive SPARCO database is the first collaborative registry in Tanzania. The registry included patients with SCD phenotypes HbSS, HbSC and HbSβ ^+^thalassemia enrolled from established sickle cell clinics in Tanzania. The establishment is similar to SPARCO Ghana and Nigeria registries (14, 15). In contrast with SPARCO Ghana, the registry enrolled patients over three regions from regional referral hospitals and district Hospitals. Patients were given a health passport after enrollment, a booklet containing information about the patient, records of the clinical visits, health education and a helpline for patients to make calls for further assistance. This makes it different from the two registries of Ghana and Nigeria. The database was firstly developed from CRF of the previous MSC and then adopted the new harmonized data elements agreed by the SickleInAfrica Consortium, which consisted of Tanzania, Ghana and Nigeria and has since grown to include Mali, Uganda, Zambia and Zimbabwe. The data elements used by the registry in Tanzania, similar to other countries in SickleInAfrica, were derived from the SCD ontology (17, 18) to support a uniform database for SCD in Africa. This registry and other sickle cell registries across the consortium were meant to establish a single consented database for SCD in Africa that is of high quality, operating through established SOPs and following the findable, accessible, interoperability and reusability (FAIR) principles, thus facilitating collaborative research across the consortium.

Our registry portrayed an opportunity to use digital revolutions to patient self-management through the use of mobile health (mhealth) with a larger sample size in contrast with published studies on the use of mhealth where the sample size was smaller (19, 20). This will improve the clinical outcome regarding drug adherence, pain management and delivery of health care services.

The growth of our registry and other registries, SickleInAfrica, will enable data to be pooled from multiple sites and increase the use of the REDCap mobile App, which becomes important for offline data collection. The harmonized data collection SOPs developed together with data elements ensure the standardization of the collected data from participating sites which constitutes the big data across the consortium, thus allowing to harness data science infrastructure to support data mining through the use of methodologies and algorisms for big data analysis and to bring insights towards a cure and better care for SCD.

Management of SCD patients requires selected medications to prolong their life. The strategy includes using folic acid to treat anemia and penicillin prophylaxis to decrease pneumococcal infections remains to be a key to reducing mortality and morbidity among patients. In our registry, only 11.7% of patients were using hydroxyurea. This is a surprising finding since the benefit of hydroxyurea in increasing HBF and improving clinical outcomes has been widely reported (21). This highlights the lack of accessibility to hydroxyurea in Tanzania, which may be related to its lack of affordability for most SCD patients (22, 23).

The standard guideline has been developed to guide SCD patients from home to tertiary hospitals and has been shared among SickleInAfrica countries. This database will ultimately lead to the improvement in evidence-based care for SCD in Tanzania (9, 14, 15). The Sickle Africa Data Coordinating Center (SADaCC) played a critical role in developing capabilities for data handling in Tanzania and across the consortium through training on data management and analysis and donations of tablets and servers.

As a result of the COVID-19 outbreak and subsequent mitigation measures in 2020, the registry was behind in reaching the target by 5%. The low number of outpatient visits, social distancing and pausing of enrollment were the major impediments during the pandemic, similar to experiences by other investigators (24, 25). Generally, the alternative enrollment methods were applied throughout the enrollment period since the fall of 2018. These included active surveillance, which involved calling known SCD patients residing in Dar es Salaam and its outskirts who were previously enrolled into the MSC to attend sickle cell clinics for enrollment into the registry, expansion of the registry to Bugando Medical Centre and Bagamoyo district hospital, community sensitization *via* mass media followed by mass screening events during the World Sickle Cell Day in June and SCD awareness events in September each year. These approaches reflected diverse strategies employed to improve enrolment into the registry, similar to the approach taken by the Kumasi-Ghana SPARCo registry (14).

Similar to the experience in Nigeria and different from Ghana, an overwhelming majority of patients enrolled in the database had HbSS compared to other SCD phenotypes HbSC and HbSβ ^+^thalassemia (14, 15, 26). Besides the expected distribution of the various SCD genotypes across Africa, this observation may also reflect a high preponderance and early onset of crisis among patients with HbSS compared to other groups, hence a higher likelihood to attend clinics early on (14, 15, 26).

Although common in all age groups, sickle cell pain episodes were reported to be < 45% in our registry, children between 7 and 13 years of age shows the highest proportional among all other age groups, while in Nigeria database > 50% reported to have pain crisis in all age group except children between age 1–5 in all age groups (15). In Ghana the pain episodes shows its peaks on the months of April and June (14) This is likely because children in this age group are in school, away from parents and guardians for long periods, and hence less likely to strictly adhere to medical advice, as previously reported (27).Our registry showed the proportion of blood transfusion frequency decreases with an increase in age from children 0–6 (33%) years to adult above 18 years of age (13.3%) in contrast with the Nigeria database which reported a steady increase in transfusion frequency from patients with 0–5 years to aged between 51–55 (15). Findings in our registry showed that at the age of 0–6 children had severe anemia (Hb g/dl <5) with 40.2% at their first visit, which is similar to the study done at MNH using the MSC data which shows slightly increase of Haemoglobin level as age increases and that could be the cause of admission as also reported to our registry together with other factors including pain and fever (7).

Our registry enrollment experienced fewer patients with clinical events since the start of 2017. Only 27.6% were covered with health insurance during their first visit to SPARCO clinics in our database which undoubtedly provide smaller opportunity to poor and disadvantaged group who had chronic diseases in Tanzania which was also reported in terms of structural and operation in other community-based health insurance schemes, community based funds and health financing studies (28–30). The major challenge for patients is the cost of the extra full blood picture diagnostic test required after clinical consultations, which needs patients to have health insurance or pay cash for further investigations.

Besides enrolment in numbers, data quality is also a paramount aspect of a good registry. Of the three thousand eight hundred (3,800) patients enrolled, some had missing responses on variables at the time of enrolment such as intercurrent medication, including 438 on status of use of hydroxyurea, 27 penicillin prophylaxis, three folic acid, 27 anti-malaria prophylaxis and ten pneumococcal vaccines during their first visit. Inadequate data collection in registries was also reported as a concern in another study in the Netherlands, where among 2,391 registered patients, the data for only 98.2% were analyzable after verification (31). Active calling of patients through mobile phones after the clinic to fill missing gaps in the CRF and during follow-up visits were the measures taken to ensure the quality of registry data.

## 5 Limitation

Since the main goal of this project was enrolling SCD patients into the registry, most of the data elements that involved laboratory investigations were not recorded in the database. This was also partly due to the inability of the patients to pay for the costs of laboratory investigations and underscores the importance of encouraging patients to enroll in health insurance plans (32). Our registry caters to patients enrolled from SPARCo-established clinics for SCD firom three regions n Tanzania. Our experiences can serve as a catalyst to inspire other health facilities across the country to initiate SCD services to join efforts to reduce the morbidity and mortality due to SCD in Tanzania and Africa.

## 6 Conclusion

Tanzania has demonstrated success in establishing the SPARCO registry, with the potential to expand to other regions in Tanzania. The SPARCO registry will improve healthcare for SCD in Tanzania and Africa through the facilitation of collaborative data-driven research for SCD.

## Figures and Tables

**TABLE 1:** Demographic characteristics of patients enrolled in the Tanzania sickle cell registry between 2017 and 2021 compared per age groups.

	N		Frequencies (%)		Age Categories			P-Value
				0–6	7–13	14–17	≥18	
***Gender***	N=3,800							
*Male*			1946 (51.2%)	798 (41.0%)	642 (33.0%)	212 (10.9%)	294 (15.1%)	<0.001
*Female*			1854 (48.8%)	638 (34.4%)	581 (31.3%)	224 (12.1%)	411 (22.2%	
***SCD-Phenotype***	N=3,800							
*SCD-SS*			3762 (99.0%)	1414 (37.6%)	1216 (32.3%)	431 (11.5%)	701 (18.6%)	0.11
*SCD-SC*			3 (0.1%)	3 (100.0%)	0 (0.0%)	0 (0.0%)	0 (0.0%)	
SCD-S *beta-plus thalassemia*			35 (0.9%)	19 (54.3%)	7 (20.0%)	5 (14.3%)	4 (11.4%)	
*Hospital name*	N=3,800							
*Amana Regional Referral Hospital*			790 (20.9%)	255 (32.3%)	254 (32.2%)	78 (9.9%)	203 (25.7%)	<0.001
*Bagamoyo District Hospital*			132 (3.5%)	43 (32.6%)	55 (41.7%)	14 (10.6%)	20 (15.2%)	
*Bugando Medical Centre*			669 (17.6%)	325 (48.6%)	238 (35.6%)	63 (9.4%)	43 (6.4%)	
*Muhimbili National Hospital/Mloganzila*			105 (2.8%)	43 (41.0%)	27 (25.7%)	10 (9.5%)	25 (23.8%)	
*Muhimbili National Hospital/Upanga*			489 (12.9%)	176 (36.0%)	181 (37.0%)	64 (13.1%)	68 (13.9%)	
*Mwananyamala Regional Referral Hospital*			604 (15.9%)	175 (29.0%)	173 (28.6%)	79 (13.1%)	177 (29.3%)	
*Temeke Regional Referral Hospital*			1011 (26.6%)	419 (41.4%)	295 (29.2%)	128 (12.7%)	169 (16.7%)	
*Management*								
*Hydroxyurea*	No	N=3,358	3,317 (88.3%)	1283 (38.7%)	1035 (31.2%)	379 (11.4%)	620 (18.7%)	<0.001
	Yes		441 (11.7%)	137 (31.1%)	178 (40.4%)	54 (12.2%)	72 (16.3%)	
*Penicillin V*	No	N=3,773	2634 (69.8%)	344 (13.1%)	1175 (44.6%)	425 (16.1%)	690 (26.2%)	<0.001
	Yes		1139 (30.2%)	1084 (95.2%)	39 (3.4%)	10 (0.9%)	6 (0.5%)	
*Folic Acid*	No	N=3,797	21 (0.6%)	11 (52.4%)	5 (23.8%)	0 (0.0%)	5 (23.8%)	0.22
	Yes		3776 (99.4%)	1,424 (37.7%)	1217 (32.2%)	436 (11.6%)	699 (18.5%)	
*Ant-Malaria Prophylaxis*	No	N=3,777	3729 (98.7%)	1,397 (37.5%)	1,201 (32.2%)	433 (11.6%)	698 (18.7%)	<0.001
	Yes		48 (1.3%)	29 (60.4%)	17 (35.4%)	2 (4.2%)	0 (0.0%)	
*Pneumococcal Vaccination Up to date*	No	N=3,790	610 (16.1%)	73 (12.0%)	38 (6.2%)	19 (3.1%)	480 (78.7%)	<0.001
	Yes		3180 (83.9%)	1358 (42.7%)	1181 (37.1%)	417 (13.1%)	224 (7.0%)	

**TABLE 2:** Clinical events of patients enrolled in the Tanzania sickle cell registry between 2017 and 2021 compared per age group.

Clinical Events		Frequencies (%)	Age Categories				P-value
			0–6	7–13	14–17	≥18	
*Did you experience any pain since the last visit? (N = 900)*	No	860 (95.6%)	337 (39.2%)	298 (34.7%)	91 (10.6%)	134 (15.6%)	0.052
	Yes	40 (4.4%)	7 (17.5%)	18 (45.0%)	6 (15.0%)	9 (22.5%)	
*Have you been admitted since the last visit? (N = 900)*	No	889 (98.9%)	341 (38.4%)	313 (35.2%)	96 (10.8%)	139 (15.6%)	0.319
	Yes	11 (1.1%)	3 (27.3%)	3(27.3%)	1 (9.1%)	4 (36.4)	
*Have you received a blood transfusion since the last visit? N = 900*	No	885 (98.3%)	339 (38.3%)	312 (35.2%)	93 (10.5%)	141 (15.9%)	0.257
	Yes	15 (1.7%)	5 (33.3%)	4 (26.7%)	4 (26.7%)	2 (13.3%)	
*Haemoglobin level (N = 900)*	Severe anemia (<5 Hb g/dl)	405 (45.0%)	163 (40.2%)	129 (31.9%)	43 (10.6%)	70 (17.3%)	0.049
	Moderate anemia(5–8g/dl)	408(45.3%)	151 (37.0%)	162 (39.7%)	43 (10.5%)	52 (12.7%)	
	Mild anemia (>8 Hb g/dl)	87 (9.7%)	30 (34.5%)	25 (28.7%)	11 (12.6%)	21 (24.1%)	
*Health Insurance (N = 900)*	No	652 (72.4%)	241 (37.0%)	229 (35.1%)	73 (11.2%)	109 (16.7%)	0.491
	Yes	248 (27.6%)	103 (41.5%)	87 (35.1%)	24 (9.7%)	34 (13.7%)	

## Data Availability

The raw data supporting the conclusions of this article will be made available by the authors, without undue reservation.
